# First Draft Genome Assembly of the Seaweed *Sargassum fusiforme*

**DOI:** 10.3389/fgene.2020.590065

**Published:** 2020-10-23

**Authors:** Shengqin Wang, Lidong Lin, Yijian Shi, Weiguo Qian, Nan Li, Xiufeng Yan, Huixi Zou, Mingjiang Wu

**Affiliations:** ^1^National and Local Joint Engineering Research Center of Ecological Treatment Technology for Urban Water Pollution, Wenzhou University, Wenzhou, China; ^2^College of Life and Environmental Science, Wenzhou University, Wenzhou, China; ^3^Dongtou Fisheries Science and Technology Research Institute, Wenzhou, China; ^4^College of Electrical and Electronic Engineering, Wenzhou University, Wenzhou, China

**Keywords:** *sargassum fusiforme*, genetic breeding, brown algae, phaeophyceae, sargassaceae

## Introduction

*Sargassum fusiforme* is a major edible brown macroalga grown in lower intertidal zones along coastlines. The macroalga is cultivated in Japan, Korea, and China (Ma et al., 2017). *S. fusiforme* is a source of bioactive compounds, such as polysaccharides, which have various functions, including antioxidants, anti-aging, memory improvement and immune regulation (Chen et al., 2016; Bogie et al., 2019; Zhang et al., 2020). It is also an essential herb used to disperse phlegm in traditional Chinese medicine (Li and Wei, 2002). However, genomic and genetic resources for this economically important seaweed are limited. This limitation could be attributed to, in part, the high levels of polysaccharides in the brown algae, which complicate DNA extraction. Recent research has shown that *S. fusiforme* contains potentially toxic quantities of inorganic arsenic, although the levels differ significantly between different growing areas (Yokoi and Konomi, 2012). Notably, a study on the molecular genetics and genomics of *S. fusiforme* could give a clue on strategies for reducing the levels of inorganic arsenic.

The genus *Sargassum* (Fucales, Phaeophyceae) consists of over 300 species of multicellular organisms, which are widely distributed in marine habitats. Many of these species are essential in the ecosystem and are economically important marine crops. For example, *Sargassum horneri* is the dominant golden-tide seaweed in the ocean (Liu et al., 2018). Despite its wide distribution, there are no available complete or draft genomes for any of the *Sargassum* genus microalgae to date. Besides, the entire Phaeophyceae class only has six decoded complete or draft genome sequences of brown algae species. These species include *Ectocarpus siliculosus* (Cock et al., 2010), *Saccharina japonica* (Ye et al., 2015), *Macrocystis pyrifera* (https://www.ncbi.nlm.nih.gov/genome/?term=Macrocystis+pyrifera), *Nemacystus decipiens* (Nishitsuji et al., 2019), *Cladosiphon okamuranus* (Nishitsuji et al., 2016), and *Undaria pinnatifida* (Shan et al., 2020). The availability of genomic resources for these essential algae will assist future selective breeding programs and fundamental genomic and evolutionary studies, among other roles.

The complete chloroplast and mitochondrial genome sequences of *S. fusiforme* have been decoded and phylogenetically analyzed (Liu et al., 2016; Yonghua et al., 2019). Recently, genome assembly using a sequencing technology that combines PacBio and Ilumina reads has shown high performance. In this study, *de novo* whole-genome sequencing in a strain of *S. fusiforme* was performed using PacBio and Illumina reads. The PacBio long reads were assembled using wtdbg2, and the Illumina short reads were used to correct the primary assembly by Pillon (Walker et al., 2014; Ruan and Li, 2020). To our knowledge, this is the first reported genome assembly in a member of the Fucales order.

### Value of the Data

The genomic sequence data generated in the present study provides essential information for further *S. fusiforme* studies, such as functional genomic analysis and genome-assisted breeding. Since these results offer the first reference genome of the order Fucales, they can be used in evolutionary studies in the Chromista kingdom. Further, the draft genome can be used as a genomic reference for the discovery of functional products and other genome mining applications.

## Materials and Methods

### Materials and DNA Extraction

The *S. fusiforme* strain used for genome sequencing in this study was collected from the Wenzhou Dongtou District, Zhejiang Province of China. Dozens of picked vesicles were cleaned in ddH_2_O, then frozen and stored in liquid nitrogen for genomic DNA extraction. The genomic DNA was extracted by the cesium chloride density gradient centrifugation method (Jagielski et al., 2017). DNA quantitation and quality checks were performed using a NanoDrop 2000 microspectrophotometer, a Qubit fluorometer, and 1% agarose gel electrophoresis (Novogene Corporation, Beijing, China).

### Illumina Sequencing and Genome Size Estimation

The isolated genomic DNA was subjected to short-read library preparation with stranded Illumina paired-end protocols (Illumina Inc., San Diego, CA, USA). Subsequently, libraries with insert lengths of 500 bp were constructed and sequenced by the Illumina NovaSeq platform. The raw reads were processed with the fastp program with the following parameters: -g -q 5 -u 50 -n 15 -l 150 (Chen et al., 2018), to obtain more than 197.5 million paired-end clean reads. The kmer frequencies (*k* = 21) were calculated using Jellyfish v2.2.10 (Marçais and Kingsford, 2011). The kmer-based statistical method from Genomescope (Vurture et al., 2017), predicted the *S. fusiforme* haploid genome length to be ~ 450 MB, with heterozygosity of ~1.0%. Notably, this estimated size is larger than the genome size of the *Sargassum* genus (196–319 Mb) previously estimated in three other species by static microspectrophotometry (Phillips et al., 2011).

### PacBio Sequencing and Genome Assembly

For long-read sequencing, the isolated genomic DNA was subjected to a large insert library preparation using the PacBio Sequel sequencing platform (PacBio, Menlo Park, CA, USA). A total of ~16 GB of polymerase reads were obtained following the manufacturer's instructions. Long reads were assembled by wtdbg2 (Ruan and Li, 2020). Due to wtdbg2 constructed the consensus using a fuzzy Bruijn graph and assembled raw reads without error correction, the primary assembly was realigned against the long reads using Minimap2, then polished with Racon, which does three replications(Vaser et al., 2017; Li, 2018). Finally, the Illumina paired-end clean reads were mapped onto the assembled contigs using bowtie2 and further corrected by Pilon (Langmead and Salzberg, 2012; Walker et al., 2014). To remove potential contaminants, contigs with a biased GC content (>0.6 or <0.4) were aligned to the NCBI non-redundant nucleic acid database with an *E*-value 1e-5. Then, a total of 112 contigs with the top 10 no matches to eukaryotes were regarded as contaminant contigs and removed from the final assembly. At last, 6,750 contigs were retained as the final assembly, with a total length of ~394.4 MB, which has an N50 value of ~142.1 KB (Table 1). Therefore, the estimated coverage for PacBio data and Illumina data were ~42 x and 180 x, respectively.

**Table 1:** Comparison of genome assemblies of seven brown algae.

**Species**	***E. siliculosus***	***S. japonica***	***N. decipiens***	***C. okamuranus***	***U. pinnatifida***	***M. pyrifera***	***S. fusiforme***
Total genome size (Mbp)	197	537	154	130	502.8	409.1	394.4
Scaffold N50 length (Kbp)	6,528	252	1,863	418	16,510	–	–
Contig N50 length (Bp)	–	58,867	6,265	21,705	1,707,374	2,573	142,085
Number of scaffolds	30	13,327	685	541	114	–	–
Number of contigs	–	–	411,597	31,858	515	246,830	6,750
Length of longest contig (Kbp)	324	466	–	–	7,337	110	931
Length of shortest contig (Bp)	–	–	–	–	–	280	319
Length of longest scaffolds (Kbp)	18,946	1,469	–	–	32,303	–	–
Length of shortest scaffolds (Bp)	869,870	500	–	–	1,000	–	–
GC contents (%)	56	49.6	54	54	50.14	50	48.4
The identified complete BUSCOs (%)	–	–	–	–	82.9	–	90.4

### Repeat Region Prediction

A *de novo* repeat library for *S. fusiforme* was conducted using RepeatModeler (Flynn et al., 2020), which employs three repeat-finding methods; RECON (Bao and Eddy, 2002), RepeatScout (Price et al., 2005), and TRF (Benson, 1999). The repeat library was then subjected to RepeatMasker to find and mask homologous repeats in the assembled genome using rmblast as the default search engine. Finally, the total length of repetitive sequences was ~239.4 Mb, accounting for ~60.7% of the draft genome size.

### Gene Prediction and Annotation

**Figure 1:**
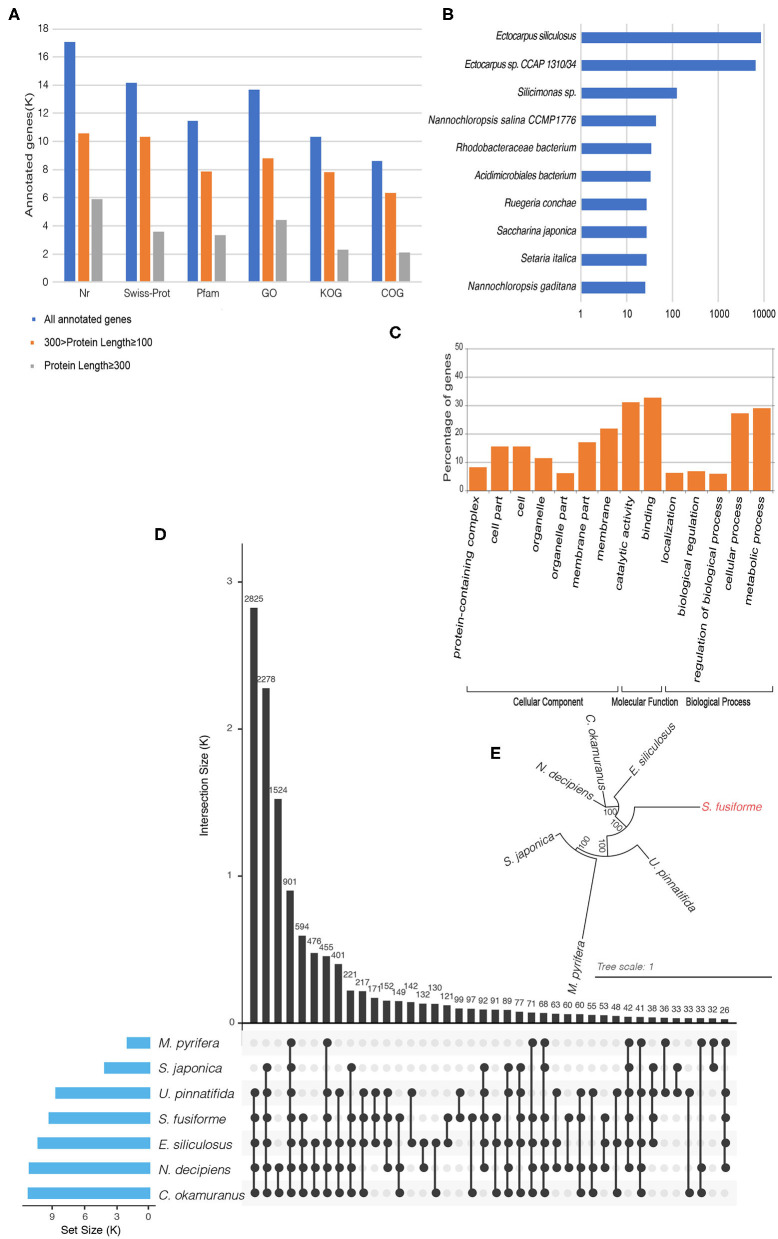
**(A)** Statistics of gene functional annotations in the *S. fusiforme* genome. **(B)** Species distribution in Nr homolog. **(C)** Gene Ontology classification. Visualization of gene ontology terms (gene number > 1,000) for the putative genes. **(D)** UpSet plot showing ortholog intersections across the seven brown algae. **(E)** Phylogenetic tree of seven brown algae using the maximum likelihood method.

The BRAKER1 pipeline was used to perform gene prediction by integrating *ab initio* gene prediction and RNA-seq based prediction, which combined the advantages of both GeneMark-ET and AUGUSTUS (Hoff et al., 2016). Raw RNA-seq data from three independent transcriptomic projects were used for gene prediction: ERR2041176, SRR12206544, and SRR5357673 (One Thousand Plant Transcriptomes Initiative, 2019). Transcript alignment was performed on the repeat masked assembly based on Tophat2, with default parameters (Kim et al., 2013). The aligned results, combined with transcript data, were used to generate initial gene structures using the GeneMark-ET tool (Lomsadze et al., 2014). The initial gene structures were used for training by AUGUSTUS to produce the final gene predictions (Stanke and Waack, 2003). Finally, 20,222 putative genes were detected. Of these putative genes, 84.45% genes had Nr homologs, 69.93% had Swiss-Prot homologs, 56.57% had Pfam homologs, 67.65% had gene ontology (GO) homologs, 51.11% had KOG homologs, and 42.60% had COG homologs (Figure 1A). In Nr homolog analysis (with an *E*-value of 0.0001), the best hit species with 15,284 genes belong to the *Ectocarpus* genus (Figure 1B). Here, the GO term distribution of the putative genes into the molecular function, cellular component, and biological process categories (Figure 1C) was determined by the Blast2GO suite with an *E*-value of 0.0001 and visualized with the WEGO 2.0 web tool at the macro-level (Conesa and Götz, 2008). COG and KOG analyses were performed with an *E*-value of 0.001 by the WebMGA server (Wu et al., 2011). KAAS web services were used to map the putative *S. fusiforme* genes onto the KEGG metabolic pathways along with genes in other plant species, including *Amborella trichopoda, Chlamydomonas reinhardtii, Ostreococcus lucimarinus, Ostreococcus tauri, Micromonas commoda, Cyanidioschyzon merolae, Galdieria sulphuraria*, and *Arabidopsis thaliana*. A total of 3,796 genes had homologs in the KEGG database, of which 1,140 were mapped onto 119 enzymes listed in the pathway categorized “Metabolism.”

### Completeness and Accuracy of the Assembly

The predicted genes from the BRAKER1 pipeline were subject to BUSCO version 3.0.2 to evaluate the completeness of the assembled genome, based on the eukaryota_odb9 database. More than 90% complete BUSCOs were detected at the protein level, with the single-copy, duplicated, fragmented, and missing accounting for 82.8, 7.6, 6.3, and 3.3%, respectively. The accuracy of the genome assembly was evaluated by mapping the clean reads against the genome with bowtie2. Finally, more than 90.7% of reads were remapped.

### Comparative Genomics

Ortholog analysis was conducted using OrthoMCL v2.0.9 based on protein datasets from the BRAKER1 pipeline and six other brown algae species: *E. siliculosus, S. japonica, M. pyrifera, N. decipiens, C. okamuranus*, and *U. pinnatifida*. Here, the protein sequences for *S. japonica* were determined by combining transcriptome data from the 1 KP (One Thousand Plant Transcriptomes Initiative, 2019), and the protein sequences for *M. pyrifera* were called using FRAGGENESCAN from pyrosequencing technology data (Konotchick et al., 2013). CD-HIT was used to remove redundant sequences (90% identity or more) in each organism. Then, non-redundant protein sequences were subjected to all-again-all Blastp with an *E*-value of 1e-5. Finally, a total of 1,4819 groups were identified by OrthoMCL and ortholog intersections across the seven brown algae, as shown in Figure 1D. Subsequently, 2,287 groups were identified as one to one orthologs from these species. These sequences were concatenated into a supergene and used for multiple sequence alignment by MAFFT. Then, a phylogenetic tree was built by PhyML (Figure 1E) using the maximum likelihood method.

## Data Availability Statement

The data used to support the findings of this study are available from Short Read Archive (SRA) database (http://www.ncbi.nlm.nih.gov/sra/) under the accession number: PRJNA597239. The raw SMRT sequencing data is available in NCBI SRA database with accession number: SRR12124361. Genome assembly and annotation data has been deposited at Figshare repository (https://figshare.com/s/517f180deb56a5b31b14).

## Author Contributions

LL, XY, HZ, and MW conceived the project and designed the objectives. LL, WQ, and NL collected the specimens. WQ and NL perform DNA/RNA extraction and sequencing. SW and YS implemented genome assembly and data analysis. SW drafted the manuscript. All authors edited and contributed to the article.

## Conflict of Interest

The authors declare that the research was conducted in the absence of any commercial or financial relationships that could be construed as a potential conflict of interest.
